# Tracing the Evolution of the *SEPALLATA* Subfamily across Angiosperms Associated with Neo- and Sub-Functionalization for Reproductive and Agronomically Relevant Traits

**DOI:** 10.3390/plants11212934

**Published:** 2022-10-31

**Authors:** Ludovico Dreni, Cristina Ferrándiz

**Affiliations:** Instituto de Biología Molecular y Celular de Plantas (IBMCP), Consejo Superior de Investigaciones Científicas-Universidad Politécnica de Valencia, 46022 Valencia, Spain

**Keywords:** MADS-box, SEPALLATA, phylogeny, core eudicots, monocots, angiosperms, inflorescence architecture, meristem determinacy, floral organ development, crops

## Abstract

SEPALLATA transcription factors (SEP TFs) have been extensively studied in angiosperms as pivotal components of virtually all the MADS-box tetrameric complex master regulators of floral organ identities. However, there are published reports that suggest that some SEP members also regulate earlier reproductive events, such as inflorescence meristem determinacy and inflorescence architecture, with potential for application in breeding programs in crops. The *SEP* subfamily underwent a quite complex pattern of duplications during the radiation of the angiosperms. Taking advantage of the many whole genomic sequences now available, we present a revised and expanded *SEP* phylogeny and link it to the known functions of previously characterized genes. This snapshot supports the evidence that the major *SEP3* clade is highly specialized for the specification of the three innermost floral whorls, while its sister *LOFSEP* clade is functionally more versatile and has been recruited for diverse roles, such as the regulation of extra-floral bract formation and inflorescence determinacy and shape. This larger pool of angiosperm *SEP* genes confirms previous evidence that their evolution was driven by whole-genome duplications rather than small-scale duplication events. Our work may help to identify those *SEP* lineages that are the best candidates for the improvement of inflorescence traits, even in far distantly related crops.

## 1. Introduction

Several classes of MIKC-type MADS-box TFs are essential for the specification of all floral organs, as described by the ABC model [1]. They function by forming homo- or heterodimers that, based on the quartet model, further combine into tetramers [2,3]. Unique tetrameric combinations of MADS-box TFs specify the identities of each of the floral organs (sepal, petal, stamen, carpel) and ovules, as well as floral meristem determinacy. Although there are plenty of in vitro experiments supporting the quartet model, conclusive proof is still lacking in vivo, where, although most interactions have been confirmed [4], it has not been exactly confirmed that tetramers must form, such that it cannot be excluded that simple dimers might be functional at least in some of the target genes [5,6].

Among these classes of MADS-box TFs, the so-called SEPALLATA (SEP) is the only common component of all the known functional complexes and is thus essential for the identities of all floral organs [7,8,9]. Most plant genomes encode several SEP TFs, which are often functionally redundant; hence, single mutants may display no or only a slight phenotype. However, in the absence of SEP function, flowers lose determinacy and all their organs are reverted to leaf-like structures [10], thus suggesting that all floral organs are, indeed, modified leaves, as proposed by Goethe in 1790 [11]. Although the ABC model was derived from the observation of loss-of-function mutants in *Arabidopsis thaliana* and *Antirrhinum majus* and although the possible homology of perianth organs between core eudicots, monocots and other taxa is a highly debated topic [12,13,14], the model seems to largely hold for angiosperms, albeit with some variations ([15,16] and references within). 

Most angiosperms produce flowers arranged in diversified clusters termed inflorescences [17,18], which are orchestrated by the inflorescence meristem (IM) and, eventually, by a subsequent hierarchical order of specialized reproductive meristems, such as the branch meristems (BMs) [19,20,21]. The relevant products of most crops and ornamental plants are their fruits and seeds or flowers, respectively. Therefore, the modification of inflorescence architecture is a major goal of breeding programs in crops and ornamental plants [22,23,24,25]. 

A few works conducted on distantly related angiosperms have shown that some SEP TFs have important roles not only in floral development but also in the regulation of IM function and/or of the other reproductive meristems that derive from it. For example, the *SEP* genes of tomato (*Solanum lycopersicum*) *JOINTLESS-2* and *ENHANCER-OF-JOINTLESS-2* (*J2* and *EJ2*) are two important domestication loci for jointless pedicel and large calyx traits, respectively, but are also important regulators of inflorescence complexity and productivity [26,27]. The loss of *OsMADS34/PANICLE PHYTOMER2* *(PAP2*) function profoundly alters inflorescence development and architecture in rice (*Oryza sativa*) [28,29,30]. Similar *SEP* genes have been shown to regulate IM function and determinacy even in the highly modified and specialized capitulum inflorescence of Asteraceae [31]. 

Within MIKC-type MADS-box genes, *SEP* forms a well-defined subfamily specific to and ubiquitous in angiosperm plants. It is divided into two major sister clades, *SEP3* (*AGL9*) and *LOFSEP* (*AGL2/3/4*) [32,33], whose split coincided with the whole-genome duplication ‘Epsilon’ (WGD-ε) that predated the most recent common ancestor (MRCA) of angiosperms [34,35]. 

Thanks to the incessant advances in DNA sequencing techniques, thousands of high-quality genomes, and even some pangenomes, are now available for most angiosperm clades, with some agriculturally important families, such as Poaceae in monocots and Solanaceae, Asteraceae, Rosaceae, Cucurbitaceae, Fabaceae and Brassicaceae in core eudicots, being particularly well-represented. The use of annotated high-quality genomes allows more precise assessment of the real numbers of orthologous genes in each species and better tracing of their patterns of duplication, loss and retention and in- and out-paralogous relationships throughout different plant taxa. The MADS-box genes encoding for subunits of tetrameric complexes are supposed to be needed in relatively strict stoichiometric ratios, which might explain why they mostly duplicate by WGDs, while copies originated from segmental or single-gene duplications are preferentially lost ([36] and references therein). This makes it effective to support their sequence-based phylogenies also by studying microsynteny, that is, the conservation of local gene content and order. Despite microsynteny having rarely been studied in plant MADS-box genes so far, such analyses have already contributed substantially to the reconstruction of their evolution and expansion in angiosperms [35,37,38]. 

Here, we present an updated analysis of *SEP* subfamily evolution in core eudicots and core monocots (i.e., Petrosaviidae *sensu* Cantino et al., 2007 [39,40]), including taxa that were previously poorly or not covered by whole genomic and transcriptomic sequencing data. In conjunction with available and future functional data, this phylogenomic snapshot helps to correlate specific *SEP* lineages with sub- and neo-functionalization processes associated with inflorescence and floral functions in non-model species and crops. 

## 2. Results and Discussion

### 2.1. Evolution of the SEPALLATA Subfamily in Core Monocots

To better understand the evolution and complexity of the *SEP* subfamily in core monocots, we took advantage of high-quality genome assemblies currently available from Poales, other commelinids and a few Asparagales (orchids, *Asparagus officinalis* and *Allium cepa*), the remaining taxonomic orders being still poorly or not represented. All the *SEP* gene models that we retrieved from these monocots, as well as those from core eudicots and *Amborella trichopoda*, had eight exons and seven introns. The few exceptions were clearly due to incomplete or incorrect annotations, showing that the *SEP* gene structure is highly conserved across angiosperms. By comparison with the protein structure of *Arabidopsis* SEP3 TF [3], we determined that, in all the *SEP* genes that we studied, the MADS-box domain is encoded by exon 1, the I (intervening) domain by exon 2, the K (keratin-like) domain by exons 3 to 6, and the less conserved C-terminal region by exons 7 and 8 (data not shown).

**Figure 1:**
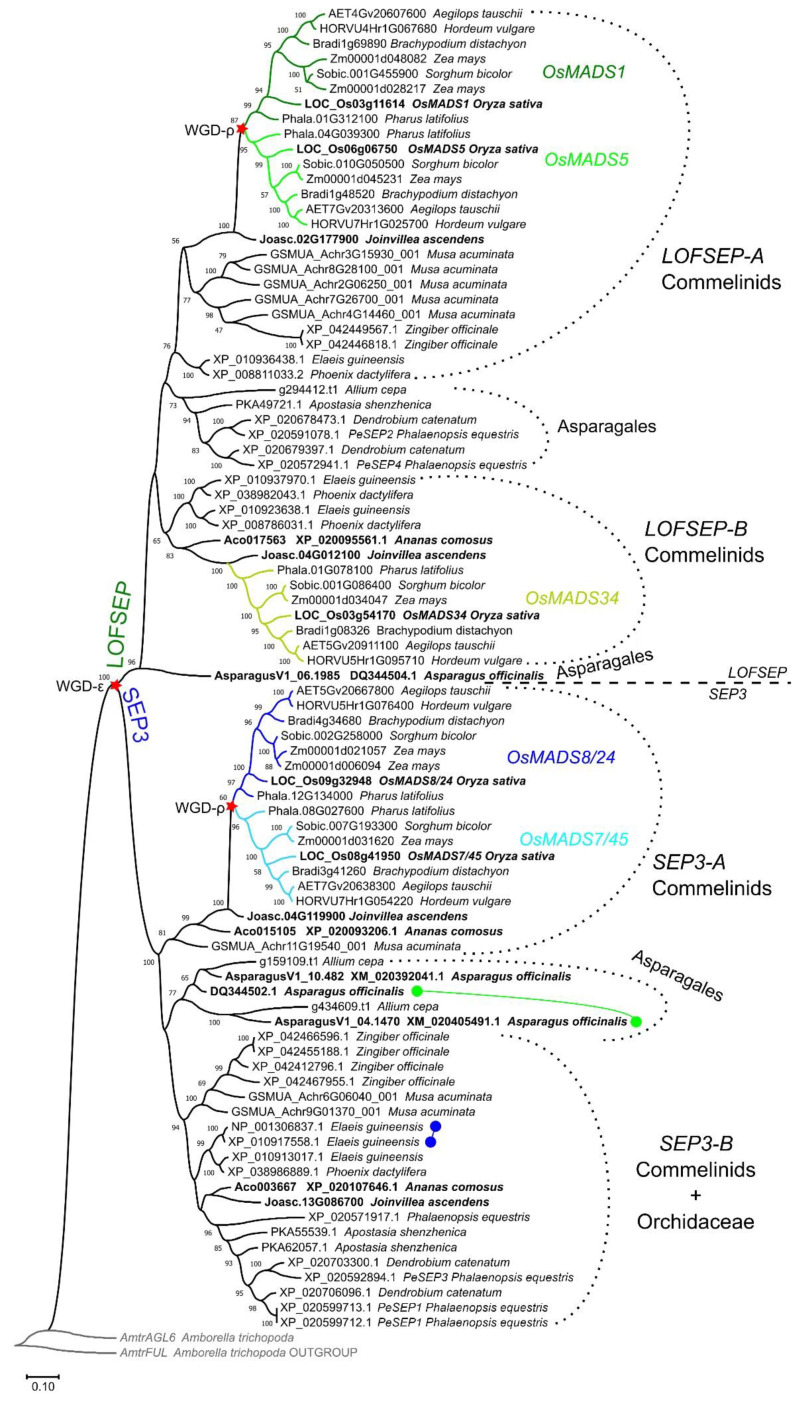
ML phylogenetic analysis of the *SEPALLATA* (*SEP*) subfamily genes from the core monocots commelinids and Asparagales. Dichotomies unequivocally linked to the angiosperm WGD-ε and the grass WGD-ρ events are marked with a red star. The three *LOFSEP* subclades of grasses, *OsMADS1*, *OsMADS5* and *OsMADS34*, are marked with different shades of green. The two *SEP3* subclades of grasses, *OsMADS7/45* and *OsMADS8/24*, are marked with different shades of blue. Two tandem duplication events of *SEP3* genes were detected in *Asparagus officinalis* and in *Elaeis guineensis*, which are marked with green and blue connected circles, respectively.

**Figure 2:**
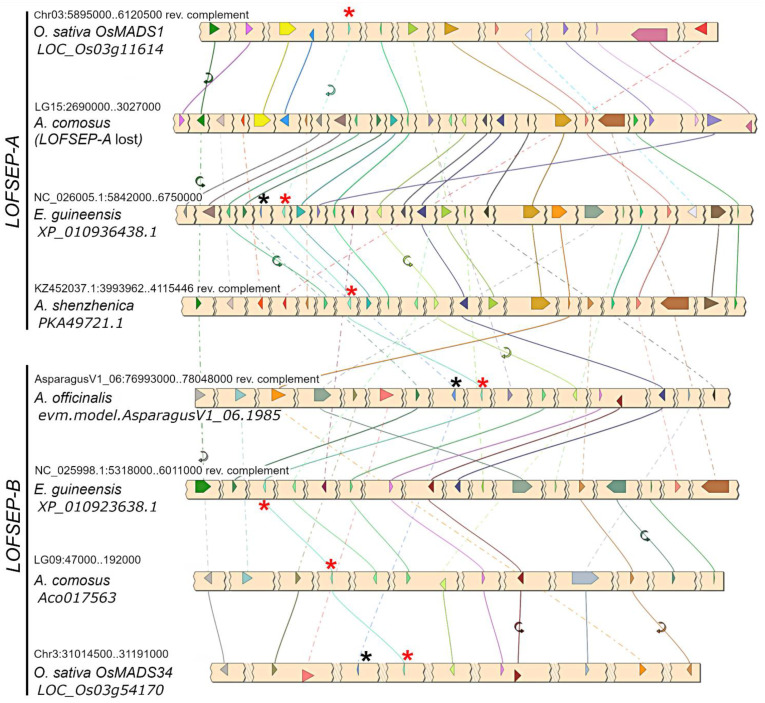
Microsynteny analysis of *LOFSEP* genes from representative species of commelinids and Asparagales. Conserved loci are connected by lines of the same color. For simplicity, the non-conserved loci were omitted. In each chromosomal region, the *LOFSEP* locus is marked with a red asterisk. The *LOFSEP-A* gene is lost in the conserved region of *Ananas comosus*, in agreement with the phylogenetic analysis shown in Figure 1. The linked *SQUA* locus, when present, is marked with a black asterisk.

A phylogenetic analysis revealed that *LOFSEP* formed two large subgroups in commelinid monocots, which we refer to as *LOFSEP-A* and *LOFSEP-B* hereafter (Figure 1). Grasses, *Joinvillea ascendens* (sister to grasses) and palms possessed genes from both clades. The result was supported by microsynteny analysis of representative species (Figure 2), which also compensated for the low bootstrap values in the phylogenetic tree for the palm species *Elaeis guineensis* and *Phoenix dactylifera*. As shown in Figure 2, a strong microsynteny is common to each group, A and B, of *LOFSEP* genes. Interestingly, a lower degree of microsynteny is also shared between A and B, indicating that they originated by an ancient large-scale or whole-genome duplication. Such an event was most likely the ancient WGD-τ that took place before the MRCA of core monocots [41,42]. Although the positions of sequences from Asparagales were unresolved in the phylogenetic tree (Figure 1), the analysis of microsynteny allowed us to assign the *LOFSEP* sequences of orchids to group A and an orphan gene of *Asparagus officinalis* (06.1985; Figure 1) to group B (Figure 2).

**Figure 3:**
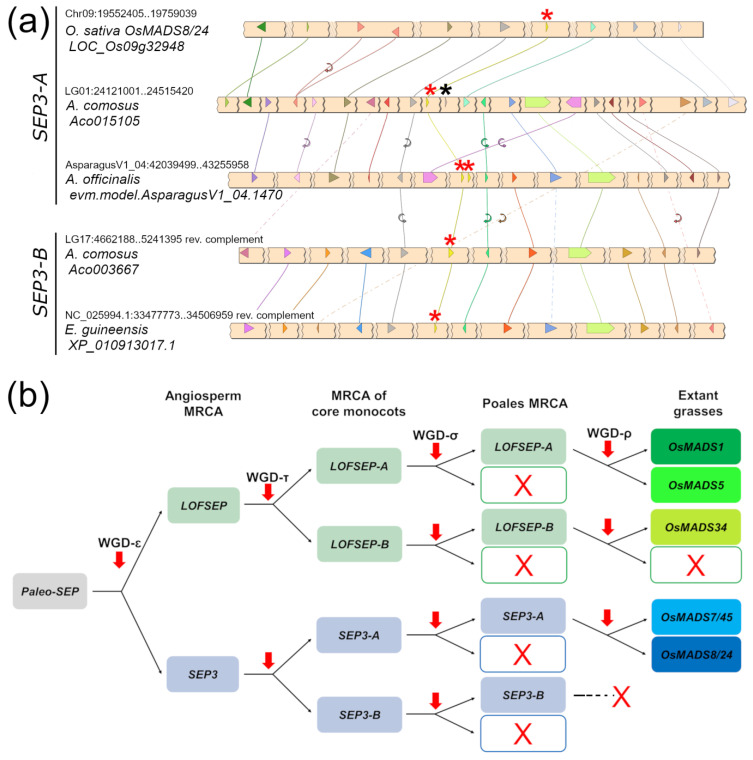
Evolutionary analysis of the *SEPALLATA* (*SEP*) subfamily in core monocots. (**a**) Microsynteny analysis of *SEP3* genes from representative species of commelinids and Asparagales. Conserved loci are connected by lines of the same color. For simplicity, the non-conserved loci were omitted. In each chromosomal region, the *SEP3* locus is marked with a red asterisk. The linked *FLC* locus, when present, is marked with a black asterisk. (**b**) Representation of the most likely pattern that drove the evolution of the *SEP* subfamily in extant grasses (Poaceae), based on our analysis and previous works. Based on the phylogeny results shown in Figure 1, the grass lineage lost *SEP3-B* after its divergence from the sister family Joinvilleaceae.

A very similar picture emerged from the analysis of the *SEP3* clade, which was also separated into two large ‘A’ and ‘B’ groups (Figure 1). In this case, all the genes from grasses fell in the *SEP3-A* group, suggesting that grasses lost *SEP3-B* after their divergence from *Joinvillea ascendens*, while all orchid genes clustered strongly with several other genes of commelinids to form the *SEP3-B* group. Pineapple (*Ananas comosus*), *Joinvillea ascendens* and banana (*Musa acuminata*) possessed genes from both clades (Figure 1). Microsynteny results further supported the existence of the two groups (Figure 3). The small unresolved clade of five *Allium cepa* and *Asparagus officinalis* genes (Figure 1) likely belong to *SEP3-A*, based on microsynteny scores (Figure 3a). As exceptions to the evidence that single-gene duplications of MADS-box genes are rapidly lost, we found tandem duplications of *SEP3* genes in *Asparagus* and *Elaeis* (Figure 1).

In conclusion, core monocots are characterized by four main groups of *SEP* genes: *LOFSEP-A*, *LOFSEP-B*, *SEP3-A* and *SEP3-B*, which, however, have been differentially retained throughout their radiation. Among the species that we analyzed, only *Joinvillea ascendens* (Poales, Joinvilleaceae) possessed member genes from all four clades (Figure 1).

#### Deciphering the Evolution of SEPALLATA Genes along the Lineage That Led to Grasses

Since the phylogenomic data suggested that an early duplication of both *LOFSEP* and *SEP3* occurred in monocots, we sought to reconstruct the subsequent evolutionary path of these genes in Poaceae, the family of true grasses and cereals. 

Two more rounds of WGD occurred in the MRCA of Poales (WGD-σ) and then in the MRCA of Poaceae (WGD-ρ) [43], which would predict up to eight *SEP3* and eight *LOFSEP* genes in extant diploid grasses, such as rice, barley (*Hordeum vulgare*), *Brachypodium distachyon*, *Sorghum bicolor* and *Pharus latifolius*. Instead, only two *SEP3* (*OsMADS7/45* and *OsMADS8/24*) and three *LOFSEP* (*OsMADS1*, *OsMADS5* and *OsMADS34*) paralogous lineages have been maintained in grasses, respectively (Figure 1, Table 1), which we named after their corresponding genes in rice [44]. These five lineages are highly conserved in diploid grasses (Figure 1, Table 1). Comparison of the relatively ancient allotetraploid maize (*Zea mays*; [45,46,47]) versus the recent allohexaploid bread wheat (*Triticum aestivum*; [48]) gives clues as to the speed of the process of selection of *SEP* homeologous genes after a polyploidization event: while only two out of five duplicated copies have been retained in maize, three homeologs for each gene still exist in bread wheat (Table 1). In addition, atypical local duplications of the *OsMADS1-* and *OsMADS5*-like genes were found in the *Aegilops*–*Triticum* complex (Table 1), whose existence and functionality are mostly supported by transcriptome assemblies publicly available in NCBI GenBank (data not shown). Therefore, these two well-studied polyploid genomes suggest that the elimination of excessive *SEP* homeologous genes is quite a long process. In recent polyploids, processes of pseudogenization and epigenetic silencing are likely to take place beforehand [49].

Grasses are devoid of *SEP3-B* genes, while their highly homologous *OsMADS7/45* and *OsMADS8/24* paralogous lineages seem to have emerged by duplication of *SEP3-A* after their divergence from Joinvilleaceae (Figure 1), suggesting that such duplication coincided with the grass-specific WGD-ρ. This is strongly supported by the observation that the *OsMADS7/45* and *OsMADS8/24* lineages reside in highly syntenic chromosomes, as can be seen, for example, in synteny maps of rice and barley [50]. Based on our phylogeny results (Figure 1), the origins of the *OsMADS1* and *OsMADS5* paralogous lineages were likely the same; however, they are located on unrelated chromosomes, and *OsMADS5* even lost the microsynteny shared by the other monocot *LOFSEP* genes (data not shown). This suggests that either *OsMADS5* transposed or that major rearrangements of its genomic position occurred in the grass MRCA. 

The third and more functionally diverged *LOFSEP* clade of grasses is *OsMADS34*, which was believed to exist only in grasses up to now. However, our analysis clarified that it belongs to the *LOFSEP-B* lineage (Figure 1 and Figure 2), meaning that it very likely diverged from its out-paralogues *OsMADS1* and *OsMADS5* at the time of the ancient WGD-τ, which occurred before the MRCA of core monocots [41,42]. Since rice *OsMADS34/PAP2* is an important regulator of inflorescence architecture [28,29,30,51], this interesting and non-canonical *SEP* function might exist also in its orthologues within and outside grasses—a matter that requires further research.

Taken together, our data support a precise pattern of *SEP* subfamily evolution and expansion in grasses, which is summarized in Figure 3b, where the Poales-specific WGD- σ made no contribution. 

In general, the rate of sequence divergence seems to be much higher in LOFSEP than in SEP3 TFs, which could already be noticed by comparing the homeologous peptides encoded by bread wheat A, B and D sub-genomes. The SEP3-like homeologous peptides accumulated just 0–4 aminoacidic changes only in the C-terminus (Figures S1–S5), suggesting that SEP3 is under stronger selective constraints.

### 2.2. Three LOFSEP Sister Clades and a Single SEP3 Clade Evolved in Core Eudicots

**Figure 4:**
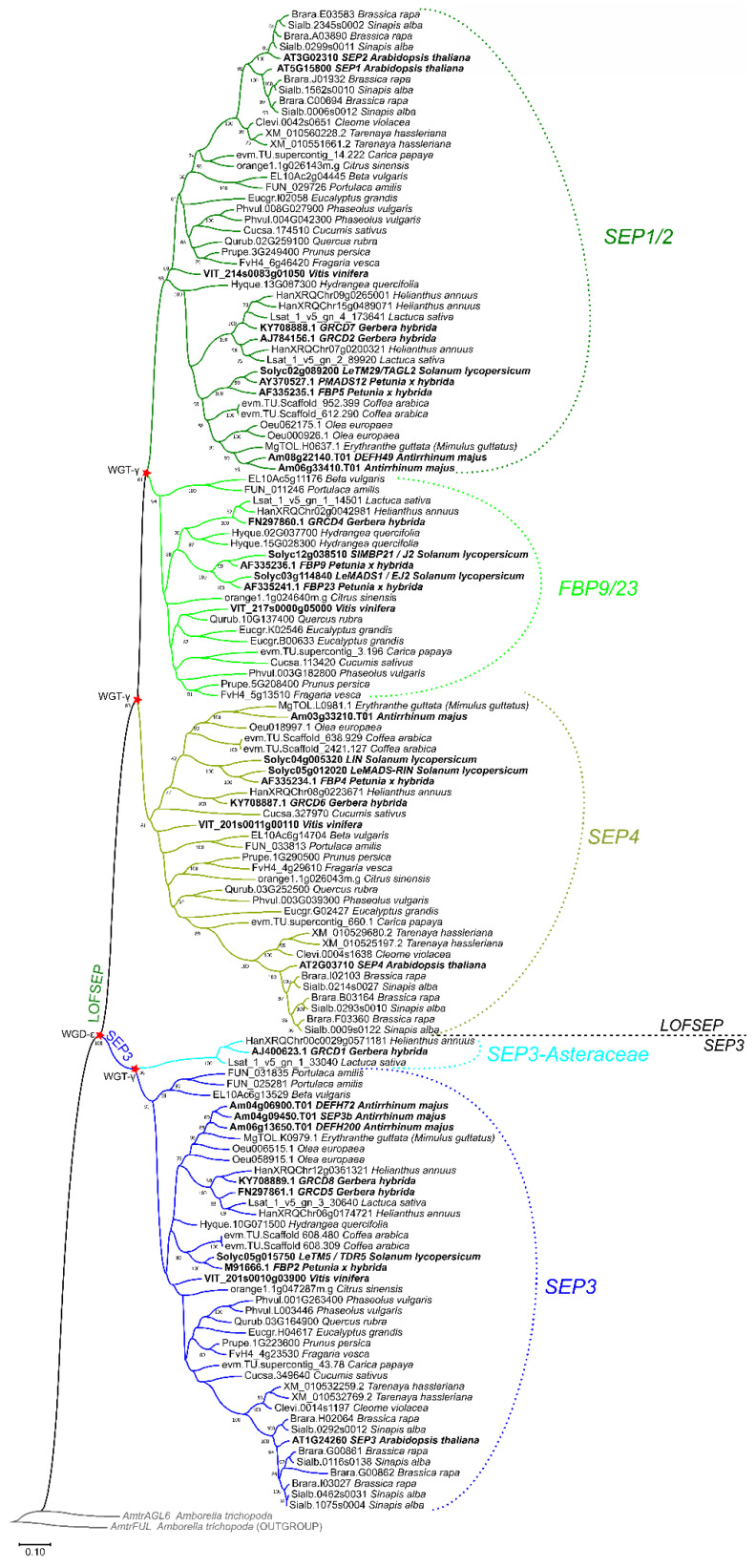
ML phylogenetic analysis of the *SEPALLATA* (*SEP*) subfamily genes in core eudicots. Dichotomies unequivocally linked to the angiosperm WGD-ε and the core eudicot WGT-γ events are marked with a red star. The three *LOFSEP* subclades of core eudicots, *SEP1/2*, *FBP9/23* and *SEP4*, are marked with different shades of green. The main *SEP3* subclade and the Asteraceae-specific *SEP3* clade are marked with different shades of blue.

**Figure 5:**
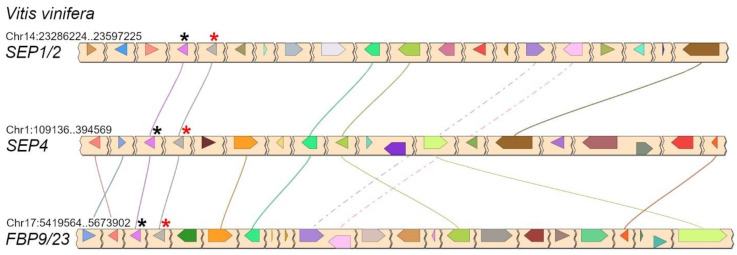
Conserved microsynteny between the three *LOFSEP* subclades of core eudicots, visualized in grape (*Vitis vinifera* L.). In each chromosomal region, the linked *LOFSEP* and *SQUA* loci are marked with red and black asterisks, respectively. They form the three tandems *SEP1/2–FL*, *SEP4–EuAP1* and *FBP9/23–EuFUL* [35].

In core eudicots, our analysis confirmed with strong support the existence of three highly conserved *LOFSEP* clades (Figure 4), which we named *SEP1/2*, *FBP9/23* and *SEP4*, in agreement with a previous work [32]. Since all extant core eudicots are descendants of a hexaploid MRCA, such expansion of the *LOFSEP* lineage is likely related to the ancestral whole-genome triplication event known as gamma (WGT-γ) [52,53,54,55,56]. Indeed, our analysis of grape (*Vitis vinifera*), a model for the study of genome evolution in core eudicots [34,55], revealed that the genomic regions of *SEP1/2*, *FBP9/23* and *SEP4* share significant collinearity with each other (Figure 5), in agreement with previous models of the origin of angiosperm-specific MADS-box subfamilies [35]. The *FBP9/23* clade was lost in Brassicales after the divergence from *Carica papaya* and, probably, also in coffee (*Coffea arabica*) and Lamiales (which are represented by *Erythranthe guttata*, *Antirrhinum majus* and *Olea europaea* in our analysis) (Figure 4). Three MADS-box genes involved in inflorescence complexity in tomato, *J2*, *EJ2* and *LONG INFLORESCENCE* (*LIN*), have been reported as *SEP4* homologues [26]. However, our phylogenetic analysis unambiguously placed *J2* and *EJ2* in the *FBP9/23* clade, while only *LIN* and its close homolog *RIPENING INHIBITOR* (*RIN*/*LeMADS-RIN*; [57]) belonged to the *SEP4* clade (Figure 4). 

In striking contrast, we only found one conserved monophyletic *SEP3* group in core eudicots, except for an Asteraceae-specific clade (Figure 4) that was already reported by Malcomber and Kellogg [32]. In the *Vitis vinifera* genome, the only *SEP3* locus resides on chromosome 1, orthologous to the whole core eudicot clade. As expected, we have identified two other microsyntenic regions in grape that derived from WGT-γ, on chromosomes 14 and 17 (data not shown), but these have lost their ancestral *SEP3* copies. Intriguingly, the genomic location of the Asteraceae-specific *SEP3* clade corresponds to the microsyntenic region of grape chromosome 17 (data not shown), revealing that this lineage has ancient origins related to WGT-γ. Considering the evolutionary position of Asteraceae, this implies that recurrent independent losses of this paralogous clade occurred a surprising number of times throughout the radiation of extant core eudicots.

**Figure 6:**
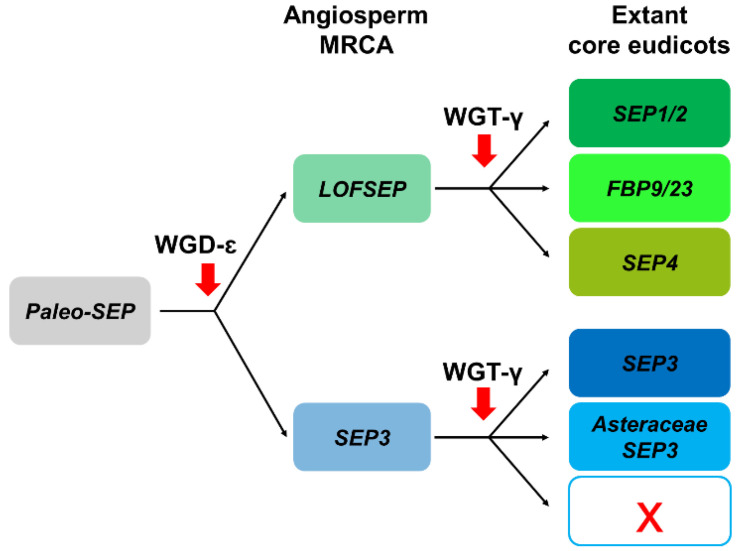
Representation of the most likely pattern that drove the evolution of the *SEPALLATA* (*SEP*) subfamily in extant core eudicots, based on our analysis and previous works.

In conclusion, the *LOFSEP* clade is significantly more expanded than *SEP3* in core eudicots, and those genomes that experienced only WGT-γ are predicted to possess a 3:1 ratio of *LOFSEP* to *SEP3* genes (Figure 4 and Figure 6), while further cycles of polyploidizations and gene losses occurred repeatedly and independently in the majority of core eudicot lineages. Our analysis supports the model of *SEP* subfamily expansion in core eudicots represented in Figure 6.

### 2.3. Conserved Genetic Linkage between SEPALLATA, SQUAMOSA and FLOWERING LOCUS C Subfamilies

Phylogenomic reconstructions showed that, in the angiosperm MRCA, the ancestral *LOFSEP* and *SQUAMOSA* (*SQUA*) genes formed a close tandem, while the ancestral *SEP3* was in tandem with *FLOWERING LOCUS C* (*FLC*), and that this configuration has been maintained in many extant angiosperms [35]. In core eudicots, *SQUA* underwent a process of triplication just as *LOFSEP* did, which led to three paralogous *LOFSEP–SQUA* tandems, as clearly shown in the grape genome (Figure 5). All these *LOFSEP–SQUA* and *SEP3–FLC* linkage relationships were lost in the lineage of Arabidopsis and other Brassicaceae. 

While noticing several such tandems during our analyses of monocots (Figure 2 and Figure 3a and additional data not shown), we found that only one *LOFSEP*–*SQUA* tandem, i.e., *OsMADS34*–*OsMADS14*, is still conserved in rice (Figure 2), with an intergenic space of just 6 kb, and in other grasses. These two loci act synergistically in floral induction in rice [58], and the latter regulates vernalization-induced flowering in winter cereal crops [59]. An *SEP3*–*FLC* tandem is also conserved in rice genomes: *OsMADS7/45–OsMADS37* [35,44]. More generally, both *LOFSEP* and *SQUA* genes play pivotal and diversified roles in agronomically relevant traits, such as floral induction, vernalization, inflorescence architecture and flower and fruit development. Targeted gene modifications, selection of natural or mutagenesis-induced variants and functional characterizations must be carried out with awareness of these conserved genetic linkage groups, which also hint at possible coregulation mechanisms. In tomato, the misinterpretation of the classic *rin* (*ripening inhibitor*; [60]) mutant led to models depicting the *SEP4* ortholog *RIN* (Figure 4) as indispensable to the induction of fruit ripening. Unexpectedly, however, *rin* is not a knock-out but a gain-of-function mutant encoding a chimeric protein from *RIN* and from the downstream *SQUA* gene *Macrocalyx* (*MC*), whose new properties as a transcriptional repressor actively repress ripening: *RIN*, indeed, is not indispensable to the induction of fruit ripening, being only required for the completion of normal ripening [61].

### 2.4. Patterns of Sub- and neo-Functionalization Associated with Diverged SEPALLATA Lineages

Our analysis provides new insights into the evolutionary history of the *SEP* subfamily in core monocots and core eudicots. Inferred polyploidization events at the base of both lineages caused a first round of independent amplifications of *LOFSEP* and *SEP3* genes, followed by many others throughout the radiation of these angiosperms. The resulting duplicated genes followed different paths of retention and loss in different taxa. In addition, *SEP* genes seem to have diverged significantly between commelinids and Asparagales, and even within Asparagales. Here, we were able to bypass the limits of phylogenetic analysis by analyzing microsynteny.

An increasing number of functional studies are clarifying that the concept of full redundancy is misleading and that the several *LOFSEP* and *SEP3* subclades that we have defined are instead specialized to regulate specific functions. *Arabidopsis* has only one *SEP3* gene (Figure 4), which is highly redundant, along with the *LOFSEP* genes *SEP1* and *SEP2*, in conferring FM determinacy and the identities of the three inner floral whorls. The *Arabidopsis sep1 sep2 sep3* triple mutant produces indeterminate flowers made only of sepals [7]. *SEP3* in not expressed at early developmental stages in the first whorl domain [62], where the last *LOFSEP* member of *Arabidopsis*, *SEP4*, is expressed instead [10]. In the *Arabidopsis sep1 sep2 sep3 sep4* quadruple mutant all the floral organs are converted to leaves, showing that *SEP4* alone is sufficient to specify sepal identity in the *sep1 sep2 sep3* triple-mutant background [10]. Despite the significant degree of redundancy shown under experimental conditions, mass spectrometry analysis of in vivo formed complexes showed that SEP3 is far more abundant than SEP1 and SEP2 in the petal, stamen and carpel identity MADS-box complexes of *Arabidopsis*, while SEP4 is absent [4]. Moreover, the transcriptional activation potential of SEP3 exceeds those of SEP1 and SEP2 [63]. Altogether, these data point to SEP3 as the most important SEP TF for floral identity in Arabidopsis. Unfortunately, only partial gene titration experiments on *sep* mutants have been reported so far [9,10], which include data not shown, yet which support the molecular data. Other studies have suggested that *SEP3* and *LOFSEP* are even more sub-functionalized in other species. The specific or preferential expression of *SEP3* genes in the three inner whorls is commonly observed in core eudicots (petal, stamen, gynoecium; [62,64,65,66]) and grasses (lodicule, stamen, gynoecium; [67]). Therefore, cases where *SEP3*-like genes are involved in first-whorl organ development, as in the orchid *Phalaenopsis equestris* [68], probably reflect deviations from a conserved and ancestral model which caused the expansion of *SEP3* gene activity in the outermost perianth organs or calyx. Interestingly, the sepals of *Phalaenopsis* are showy and mimic petals. *Petunia hybrida* has only one *SEP3* gene, *FLORAL BINDING PROTEIN2* (*FBP2*), whose loss of function results in the conversion of petals into green sepaloid organs and the development of secondary inflorescences in the third whorl [64,69,70]. In Asteraceae, one *SEP3* gene of *Gerbera hybrida* is *GERBERA REGULATOR OF CAPITULUM DEVELOPMENT5* (*GRCD5*; Figure 4), which also shows unique functions in petal development. Interestingly, *GRCD1* belongs to the Asteraceae-specific *SEP3* clade (Figure 4) and seems to be sub-functionalized to regulate stamen identity. In orchids, the suppression of one *SEP3* gene is also sufficient to trigger a partial loss of floral organ identity [68,71]. The co-suppression of rice *OsMADS7/45* and *OsMADS8/24* led to serious defects in all the inner three whorls (lodicule, stamen, gynoecium; [67]), suggesting that *SEP3* and *LOFSEP* are even more sub-functionalized in rice than in core eudicot model species. However, the reversion of the flower into leafy, indeterminate, shoot-like structures required the simultaneous suppression of most or all *LOFSEP* and *SEP3* genes in core eudicots and rice [10,31,67,72]. In rice, we have previously shown that the combined mutation of the three *LOFSEP* genes (*OsMADS1*, *OsMADS5* and *OsMADS34*) is sufficient to convert the flowers almost completely into indeterminate leafy organs, but it is important to notice that this phenotype was associated with a dramatic decrease in the expression of *SEP3* genes (*OsMADS7/45* and *OsMADS8/24*). Therefore, it seems that rice *LOFSEP* genes positively regulate *SEP3* genes, and that the floral phenotypes observed in *lofsep* triple mutants were caused by global reductions in both *LOFSEP* and *SEP3* function [73]. Rice *LOFSEP* genes are also important regulators of the bract- and prophyll-like spikelet organs that protect the flower and represent evolutionary innovations: *OsMADS1* specifies the identities of lemmas and paleas [74,75,76,77,78], *OsMADS34* represses the development of the two lateral sterile lemmas [28,29,79] and, finally, all these organs are converted into leaves in the *osmads1 osmads5 osmads34* triple mutant [73]. 

It is intriguing that *LOFSEP* genes have been recruited to regulate inflorescence development in several species, mostly by limiting branching and promoting the switch to FM identity, which are functions that temporally precede their well-known and essential functions in flower development. In Solanaceae, the *FBP9/23* subclade is the main player, with contributions from *SEP4* [26,27,72], while *SEP1/2* genes are the main regulators of IM determinacy in the capitulum of *Gerbera hybrida* [31,80]. Rice *OsMADS34* and *SQUA*-like genes synergistically act to specify IM identity, downstream of the florigen signal [58]. Subsequently, *OsMADS34* limits inflorescence primary branching by repressing IM activity [28,29,30]. In addition, *OsMADS34* shares functions with *OsMADS5* in repressing secondary branching by promoting the maturation of meristems toward the spikelet meristem stage and in promoting the elongation of the inflorescence rachis and branches [30]. As a consequence, *osmads34* and *osmads5 osmads34* knock-out mutants produce much more branched inflorescence primordia, but several meristems subsequently fail to develop into mature, fertile spikelets [30], similarly to what has been observed in tomato plants defective with respect to *J2*, *EJ2* and *LIN* functionality [26]. Unfortunately, mild *OsMADS34* alleles able to trigger more productive inflorescences, which could be beneficial for breeding programs, have not emerged so far. The function of *OsMADS34* in inflorescence architecture is likely conserved in other grasses [81,82]. Our analysis reveals that genes similar to *OsMADS34* exist in other core monocots, which opens new perspectives for future functional studies, especially in monocot crops with complex inflorescences, such as pineapple and palms. 

Given the fact that different *LOFSEP* subclades have been recruited for similar inflorescence functions in rice, Solanaceae and Asteraceae reveal their ancestral potential in regulating inflorescence development, which then was lost or retained during evolution.

## 3. Conclusions

We found both *LOFSEP* and *SEP3* genes ubiquitously in core eudicot and monocot species, which suggests that each clade has specific essential functions besides their shared roles in FM and floral organ identity. The strong conservation of *SEP3* genes, in terms of sequences and expression patterns, suggests that their major role in petal, stamen and carpel identity complexes was established and fixed before the MRCA of monocots + eudicots, while *LOFSEP* genes appear to have enjoyed more functional flexibility to allow their neo-functionalization, acquiring diversified roles in different angiosperm families, such as the regulation of bract identity, pedicel abscission zone, calyx size and inflorescence architecture. Therefore, besides their relevance for understanding angiosperm evolution, some *SEPALLATA* genes are major players in agronomically relevant traits. While *SEP3* or other MADS-box homeotic mutants are potentially useful in the creation of ornamental floral oddities and flowers less attractive to insect pests [71,83], the biotechnological manipulation of *LOFSEP* genes or their network shows promise with respect to the improvement of inflorescence characters, such as numbers of flowers and fruits. To this aim, genes from the *FBP9/23* and *SEP4* clades are promising candidates for further studies in asterid species with branched inflorescences, while homologues of rice *OsMADS34* are likely the main players in grasses and, perhaps, even in other core monocots. 

## 4. Materials and Methods

All the SEPALLATA genes used in this study were identified through BLAST analysis of the following databases: NCBI Genome (Tarenaya hassleriana, Gerbera hybrida, Petunia x hybrida, Zingiber officinale, Elaeis guineensis, Phoenix dactylifera, Apostasia shenzhenica, Dendrobium catenatum, Phalaenopsis equestris), Gramene (Aegilops tauschii and Triticum aestivum), www.oniongenome.wur.nl (Allium cepa), the Snapdragon Genome Database (http://bioinfo.sibs.ac.cn/Am/index.php; Antirrhinum majus) and Phytozome 13 (all the other species). Genes from Asparagus officinalis and Ananas comosus were identified from both the NCBI and Phytozome 13 databases, and incomplete or incorrect annotations were eventually corrected by searching the NCBI Transcriptome Shotgun Assembly (TSA) database. Accession numbers are available in Table 1 and Table S1. Protein sequences were aligned using MAFFT (https://mafft.cbrc.jp/alignment/server/), checked manually and then back-translated to nucleotide alignments with PAL2NAL (http://www.bork.embl.de/pal2nal/). 

Phylogenetic trees were calculated with MEGA 11 [84]. Evolutionary history was inferred using the Maximum Likelihood (ML) method and the Tamura–Nei model [85]. The model was accepted based on the high consistency of the resulting topologies with respect to previously published clades and genes. The trees with the highest log likelihoods were shown. The percentage of trees in which the associated taxa clustered together is shown next to the branches. Initial tree(s) for the heuristic search were obtained automatically by applying the Neighbor-Join and BioNJ algorithms to a matrix of pairwise distances estimated using the Tamura–Nei model and then selecting the topology with a superior log likelihood value. The trees were drawn to scale, with branch lengths measured as the number of substitutions per site. Codon positions included were 1st + 2nd + 3rd.

Microsynteny was calculated and scored using SynFind [86] on the CoGe platform (https://genomevolution.org/coge/). Then, selected genomic regions and genes were downloaded from Phytozome 13 Phytomine and NCBI Genomes. Gene homology was confirmed manually with BLAST analysis. The final images shown in this work were generated with Simple Synteny online (https://www.dveltri.com/simplesynteny/; [87]). All the databases and online tools were accessed between November 2021 and July 2022.

Images were edited with InkScape 0.92 (https://inkscape.org/) and GIMP 2.10.32 (https://www.gimp.org/).

## Figures and Tables

**Table 1:** Accessions of all the *LOFSEP* and *SEP3* loci found in the diploid genomes of *Oryza sativa* (rice), *Pharus latifolius*, *Brachypodium distachyon*, *Hordeum vulgare* (barley), *Aegilops tauschii* and *Sorghum bicolor*, in the ancient allotetraploid *Zea mays* (corn) and in the recent allohexaploid *Triticum aestivum* (bread wheat).

	*Oryza sativa*		*P. latifolius*	*B. distachyon*	*H. vulgare*	*A. tauschii*	*T. aestivum*	*S. bicolor*	*Z. mays*
*LOFSEP*	*OsMADS1*						TraesCS4A02G028100 TraesCS4A02G058900 TraesCS4A02G078700		Zm00001d028217 Zm00001d048082
		LOC_Os03g11614	Phala.01G312100	Bradi1g69890	HORVU4Hr1G067680	AET4Gv20607600 AET4Gv20611300 AET4Gv20678000	TraesCS4B02G245700 TraesCS4B02G245800 TraesCS4B02G277800	Sobic.001G455900	
							TraesCS4D02G243700 TraesCS4D02G245200 TraesCS4D02G276100		
	*OsMADS5*						TraesCS7A02G122000 TraesCS7A02G122100		
		LOC_Os06g06750	Phala.04G039300	Bradi1g48520	HORVU7Hr1G025700	AET7Gv20313600 AET7Gv20313900	TraesCS7B02G020800 TraesCS7B02G020900 TraesCS7B02G021000	Sobic.010G050500	Zm00001d045231
							TraesCS7D02G120500 TraesCS7D02G120600		
	*OsMADS34*						TraesCS5A02G391800		
		LOC_Os03g54170	Phala.01G078100	Bradi1g08326	HORVU5Hr1G095710	AET5Gv20911100	TraesCS5B02G396700	Sobic.001G086400	Zm00001d034047
							TraesCS5D02G401700		
*SEP3*	*OsMADS7/45*						TraesCS7A02G260600		
		LOC_Os08g41950	Phala.08G027600	Bradi3g41260	HORVU7Hr1G054220	AET7Gv20638300	TraesCS7B02G158600	Sobic.007G193300	Zm00001d031620
							TraesCS7D02G261600		
	*OsMADS8/24*						TraesCS5A02G286800		Zm00001d021057 Zm00001d006094
		LOC_Os09g32948	Phala.12G134000	Bradi4g34680	HORVU5Hr1G076400	AET5Gv20667800	TraesCS5B02G286100	Sobic.002G258000	
							TraesCS5D02G294500		

## Data Availability

No new data were created or analyzed in this study. Data sharing is not applicable to this article.

## Supplementary Materials

The following are available online at https://www.mdpi.com/article/10.3390/plants11212934/s1, Figure S1: Alignment of all the LOFSEP, OsMADS1-like proteins of *Triticum aestivum* (bread wheat), Figure S2: Alignment of all the LOFSEP, OsMADS5-like proteins of *Triticum aestivum* (bread wheat), Figure S3: Alignment of the three LOFSEP, OsMADS34-like homeolog proteins of *Triticum aestivum* (bread wheat), Figure S4: Alignment of the three SEP3, OsMADS7/45-like homeolog proteins of *Triticum aestivum* (bread wheat), Figure S5: Alignment of the three SEP3, OsMADS8/24-like homeolog proteins of *Triticum aestivum* (bread wheat), Table S1: Accession codes for all the genes reported in this study.
