# Supplementary material for: Tracing the Evolution of the SEPALLATA Subfamily across Angiosperms Associated with Neo- and Sub-Functionalization for Reproductive and Agronomically Relevant Traits
Source: Plants (Basel). 2022 Oct 31;11(21):2934. doi: 10.3390/plants11212934 (PMC9656651; doi:10.3390/plants11212934)
Supplement: Supplementary file 1 [file plants-11-02934-s001.zip › Figures S1-S5.pdf]

plants

MDPI

Supplementary Materials

```
4A02G02810 : MGRGKVEIRRIENKTTRQVTFTKRRNGLLKKAYELSLLCDAEVALIIFSGGGRLFEFSSSSCMYK :  65
4A02G05890 : MGRGKVEMRRIENKISRQVTFAKRRNGLLKKAYELSLLCDAEVALIIFSGRGRLFEFSSSSCMYK :  65
4A02G07870 : --------------------------------------------------------------MYK :   3
4B02G24570 : MGRGKVEMRRIENKISRQVTFAKRRNGLLKKAYELSLLCDAEVALIIFSGRGRLFEFSSSSCMYK :  65
4B02G24580 : MGRGKVEMRRIENKISRQVTFAKRRNGLLKKAYELSLLCDAEVALIIFSGRGRLFEFSSSSCMYK :  65
4B02G27780 : MGRGKVEIRRIENKTTRQVTFTKRRNGLL-KAYELSLLCDAEVALVIFSGGGRLFEFSSSSCMYK :  64
4D02G24370 : MGRGKVEMRRIENKISRQVTFAKRRNGLLKKAYELSLLCDAEVALIIFSGRGRLFEFSSSSCMYR :  65
4D02G24520 : MGRGKVEMRRIENKISRQVTFAKRRNGLLKKAYELSLLCDAEIALIIFSGRGRLFEFSSSSCMYK :  65
4D02G27610 : MGRGKVEIRRIENKTTRQVTFTKRRNGLLKKAYELSLLCDAEVALVIFSGGGRLFEFSSSSCMYK :  65

4A02G02810 : ILERYRTCNYNSPEATPPAENEINYQEYLKFKTRLEYLQSSQRNILGEDLGPLTMRELEQIENQI : 130
4A02G05890 : TLERYRTCNSNSQEAAPPLENE------------------------------------------- :  87
4A02G07870 : TLERYRTCNCNSQEATPLAENEINYQQYLKLKTRLEYLESSQRNILGEDLGPLSMKELEQIENQI :  68
4B02G24570 : TLERYRTCNSNSQEATPPLESEINYQEYLKLKTRVEFLQSSQRNILGEDLGPLSMKELDQIENQI : 130
4B02G24580 : TLERYRTCNCNSQEATPLAENEINYQQYLKLKTRLEYLESSQRNILGEDLGPLSMKELEQIENQI : 130
4B02G27780 : ILERYRTCNHNSPEATPSAENEINYQEYLKLKTRLEYLQSSQRNILGEDLGPLSMRELEQIENQI : 129
4D02G24370 : TLERYRTCNSNSQEATPPLENEINYQEYLKLKTRVEFLQSSQRNILGEDLGPLSMKELDQIENQI : 130
4D02G24520 : TLERYRTCNCNSQEATPLAENEINYQQYLKLKTRLEYLESSQRNILGEDLGPLSIKELEQIENQI : 130
4D02G27610 : ILERYRTCNYNSTEATPPAENEINYQEYLKLKTRLEYLQSSQRNILGEDLGPLSMRELEQIENQI : 130

4A02G02810 : DISLKHIRTRKNKVLLDELYDLKSKEQELLDQNKDLRKKLHDIS--CAENALHMSWQDGGHGSSS : 193
4A02G05890 : -------------------------EQELQDENKDLRKKLQDTTSSCGENAVHMSWQDGGQSSSR : 127
4A02G07870 : DISLKHIRTRKNKVLLDELYDLKSKEQELQDQNKDLRKKLQDTS--CAENALHMAWQDRGQSSSS : 131
4B02G24570 : DASLKHIRSKKNQVLLDQLFELKSKEQELQDENNDLRKKLQDTTSCCGENAVHMSWQDGGQCSSR : 195
4B02G24580 : DISLKHIRTRKNKVLLDELYDLKSKEQELQDQNKNLRKKLQDTS--CVENALHMAWQDAGQCSSS : 193
4B02G27780 : DISLKHIRTRKNKVLLHELYDLKSKEQELLDQNKDLRKKLQDIS--CAENALHMSWQDGGHGSSS : 192
4D02G24370 : DASLKHIRSKKNQVLLDQLFELKSKEQELQDENNDLRKKLQDTTSCCGDNAVHMSWQDGGQCSSR : 195
4D02G24520 : DISLKHIRTRKNKVLLDELYDLKSKEQELQDQNKNLRKKLQDTS--YAQNAPHMAWQDAGQSSSS : 193
4D02G27610 : DISLKHIRTRKNKVLLDELYDQKSKEQELLDQNKDLRRKLQDIS--CAENALHMSWQDGG----- : 188

4A02G02810 : G-HAIKTTYPGLLQRPEHDSSMQIGY-H-----QLNN-EDMAPQRLDGHLGSFAGWI------ : 242
4A02G05890 : -----------VLQHPEHDTSMQIGY-PQAYMDQLNSRDHVASERPGG--GSSAGWI------ : 170
4A02G07870 : G-HAIDTTYPGLVQHPEHDSSMQVGYNNQVYVDQPNNNEDMASQRLHG-LGTSAGWI------ : 186
4B02G24570 : ------------VLHPEHDTSMQIGY-VFVHVNQ----------------------------- : 216
4B02G24580 : G-HVIDTTYSGLVQHPEHDSSVQVGCRVRIYCNGLFIHG-------HG-SGTSRGLALRPVRS : 247
4B02G27780 : GWHAIETTYPGLLQRLEHDSSRQIGY-HQTSMDQLNN-EAMAPQCLDGHLGSTASWI------ : 247
4D02G24370 : ------------VLHPEHDTSMQIGY-PQAYMDQLNKQR-------------SRGF------- : 225
4D02G24520 : G-HVIDTTYPGLVEHPEHDSSMQVGYNNQAYVDQPNNKEDMASQRLHA-LGSSAGWI------ : 248
4D02G27610 : --HAIETTYPGLLQRPEHDSSMQIGY-HQTSMDQLNN-EDMAPQRLDGHLGSSAGWI------ : 241
```

Figure S1. Alignment of all the LOFSEP, OsMADS1-like proteins of *Triticum aestivum* (bread wheat).

```
7A02G12200 : MGRGKVELKRIDNKISRQVTFAKRRNGLLKKAYELSVLCDAEVALIIFSTRGRLFE--FSTSSCMYKTLERYRSCNFNS :  77
7A02G12210 : MGRGKVELKRIDNKISRQVTFAKRRNGLLKKAYELSLLCDAEVALIIFSARGRLFE--FSTSSRMYKTLERYRSCNFNS :  77
7B02G02080 : MGRGKVELKRIDNKSSRQVTFAKRRNGLLKKAYELSVLCDAEVALIIFSTRGRLFE--FSTSSCMYKTLERYRSCNFNS :  77
7B02G02090 : MGRGKVELKRIDNKVSRQVTFAKRRNGLLKKAYELSVLCDAVVALIIFSTRGRLFE--FSTSSYM-------------- :  63
7B02G02100 : ----------------------------------------------------------------MYKTLERYRSCNFNS :  15
7D02G12050 : MGRGKVELKRIDNKISRQVTFAKRRNGLLKKAYELSVLCDAEVALIIFSTRGRLFE--FSTSSCMYKTLERYRSCNFNS :  77
7D02G12060 : MRRGKVELKRIDNKSSRQVTFAKRRNGLLKKAHELSVLCDAEVALIIFSTRGRLFE--FSTSSCMYKTLERYRSCNFNS :  77

7A02G12200 : EATATPETEQSNYQEYLKLKTRVEFLQTTQRNHLGEDLGPLNMKELEQLENQIEISLKHIRATKSQQSLDQLFELKRKE : 156
7A02G12210 : EATATPETELSNYQEYLKLKTRVEFLQTTQRNLLGEDLGPLNMKELEQLENQIEISLKHIRATKSQQSLDQLFELKRKE : 156
7B02G02080 : EATAAPETELNNYQEYLKLKTRVEFLQTTQRNLLGEDLGPLNMKELEQLENQIEISLKHIRATKSQQSLDQLFELKRKE : 156
7B02G02090 : ------------------------------------------------------------------------------- :   -
7B02G02100 : EATSTPESEESSYQEYLKLKTRVDFLQTTQRNLLGEDLGPLNMKELEQLENHIEMSLKHIRATKSQQSFDQLFELKRKE :  94
7D02G12050 : EATAAPEIELSNYQEYLKLKTRVEFLQTTQRNLLGEDLGPLNMKELEQLENQIEISLKHIRATKSQQSLDQLFDLKRKE : 156
7D02G12060 : EATATPEPELSGYQEYLKLKQRVEFLQTTQRNLLGEDLGPLNMKELEQLENHVEISLKHIRATKSQQSFDQLLELKRKE : 156

7A02G12200 : QQLQDVNKDLRKKIQETSVENVLQMSCQ--DVGPSGSSGHANQANQQEYFHHDCDPSLHIGYQRNFLDQLNKE : 227
7A02G12210 : KQLQDVNKDLRKKIQETGADSVLQMFCQ--DVGPSGSSGHANQANQQEYFHPDCDPSLRMGYDHAYLDHLNKE : 227
7B02G02080 : QQLQDVNKDLRKKIQETSAENVLQMSCQ--DVGPSGSSGHANQANQQQHFHPACDPSMRIGYQRNFLDQLNNE : 227
7B02G02090 : ------------------------------------------------------------------------- :   -
7B02G02100 : QQLQDVNKDLRKKIQETSAESVLQMFCQDVDVGPSGSSGHANQANQQQHFHPDCDPSLRMWYDHAYLDHLNKE : 167
7D02G12050 : QQLQDVNKDLRKKIQETTAQNVLQMSCQ--DVGPSGSSGHANQANQQEHFHPACDPSLRIGYQRNFLDQLNKE : 227
7D02G12060 : QKLQDVNKDLRKKIQETSAESVLQMFYQ--DDGPSGSSGHANQANQQQHVHPDCDPSLRMWYDHAYLDHLNKE : 227
```

Figure S2. Alignment of all the LOFSEP, OsMADS5-like proteins of *Triticum aestivum* (bread wheat).

```
5A02G39180 : MGRGKVVLQRIENKISRQVTFAKRRNGLLKKAYELSLLCDAEVALVLFSHAGRLYQFSSSSNMLKTLEKYQRYIFASQDAA :  81
5B02G39670 : MGRGKVVLQRIENKISRQVTFAKRRNGLLKKAYELSLLCDAEVALVLFSHAGRLYQFSSSSNMLKTLERYQRYIFASQDAA :  81
5D02G40170 : MGRGKVVLQRIENKISRQVTFAKRRNGLLKKAYELSLLCDAEVALVLFSHAGRLYQFSSSSNMFKTLERYQRYIFASQDAV :  81

5A02G39180 : VPTTDEMQNNYLEYMELKSRVEVLQRSQRNLLGEDLAPLSTIELEQLEGQVGKTLRQIRSRKTQVLLDEMCDLKRKEQILQ : 162
5B02G39670 : VPTRDEMQNNYLEYMELKARVEVLQHSQRNLLGEDLAPLSTTELDQLESQVGKTLRQIRSRKTQVLLDEMCDLKRKEQILE : 162
5D02G40170 : APTSDEMQNNYLEYMELKARVEVLQHSQRNLLGEDLAPLSTTELDQLESQVGKTLRQIRSRKTQVLLDELCDLKRKEQMLQ : 162

5A02G39180 : DANMTLKRKLGEIELEATPDPPQQPQQQQMWQGDRGVPPHTPPQPEHFFQALERYPSLQPVFRGMDVNQPPPAWMA : 238
5B02G39670 : DANMTLKRKLGEIELEATPDPPQ--QQQQMWQGDRGVPPHTPPQPEHFFQALECYPSLQPVFRGMDVNQPPPAWMV : 236
5D02G40170 : DANMTLKRKLGEIQVEATPDPQQ--QQQQMWQGDRGVPPHTPPQPEHFFQALECYPSLQPVFRGTDVNQPPPAWMA : 236
```

Figure S3. Alignment of the three LOFSEP, OsMADS34-like homeolog proteins of *Triticum aestivum* (bread wheat).

```
7A02G26060 : MGRGRVELKRIENKINRQVTFAKRRNGLLKKAYELSVLCDAEVALIVFSNRGKLYEFCSTQSMTKTLDKYQKCSYAGPETTV :  82
7B02G15860 : MGRGRVELKRIENKINRQVTFAKRRNGLLKKAYELSVLCDAEVALIVFSNRGKLYEFCSTQSMTKTLDKYQKCSYAGPETTV :  82
7D02G26160 : MGRGRVELKRIENKINRQVTFAKRRNGLLKKAYELSVLCDAEVALIVFSNRGKLYEFCSTQSMTKTLDKYQKCSYAGPETTV :  82

7A02G26060 : QNRENEQLKNSRNEYLKLKARVDNLQRTQRNLLGEDLDSLGIKELESLEKQLDSSLKHIRTTRTQHMVDQLTELQRREQMFS : 164
7B02G15860 : QNRENEQLKNSRNEYLKLKARVDNLQRTQRNLLGEDLDSLGIKELESLEKQLDSSLKHIRTTRTQHMVDQLTELQRREQMFS : 164
7D02G26160 : QNRENEQLKNSRNEYLKLKARVDNLQRTQRNLLGEDLDSLGIKELESLEKQLDSSLKHIRTTRTQHMVDQLTELQRREQMFS : 164

7A02G26060 : EANKCLRIKLEESNQVHGQQLWEHNNNVLSYERQPEVQPPMHGGNGFFHPLDAAGEPTLHIGYPPESLNSSCMTTFMPPWLP : 246
7B02G15860 : EANKCLRIKLEESNQVHGQQLWEHNNNVLSYERQPEVQPPMHGGNGFFHPLDAAGEPTLHIGYPPESLSNSCMTTFMPPWLP : 246
7D02G26160 : EANKCLRIKLEESNQVHGQQLWEHNNNVLGYERQPEVQPPMHGGNGFFHPLDAAGEPTLHIGYPPESMSNSCMTTFMPPWLP : 246
```

Figure S4. Alignment of the three SEP3, OsMADS7/45-like homeolog proteins of *Triticum aestivum* (bread wheat).

```
5A02G28680 : MGRGRVELKRIENKINRQVTFAKRRNGLLKKAYELSVLCDAEVALIIFSNRGKLYEFCSGQSMPKTLERYQKCSYGGPDTAIQN :  84
5B02G28610 : MGRGRVELKRIENKINRQVTFAKRRNGLLKKAYELSVLCDAEVALIIFSNRGKLYEFCSGQSMPKTLERYQKCSYGGPDTAVQN :  84
5D02G29450 : MGRGRVELKRIENKINRQVTFAKRRNGLLKKAYELSVLCDAEVALIIFSNRGKLYEFCSGQSMPKTLERYQKCSYGGPDTAIQN :  84

5A02G28680 : KENELVQSSRNEYLKLKARVENLQRTQRNLLGEDLGSLGIKDLEQLEKQLDSSLRHIRSTRTQHMLDQLTDLQRKEQMLCEANK : 168
5B02G28610 : KENELVQSSRNEYLKLKARVENLQRTQRNLLGEDLGSLGIKDLEQLEKQLDSSLRHIRSTRTQHMLDQLTDLQRKEQMLCEANR : 168
5D02G29450 : KENELVQSSRNEYLKLKARVENLQRTQRNLLGEDLGSLGIKDLEQLEKQLDSSLRHIRSTRTQHMLDQLTDLQRKEQMLCEANK : 168

5A02G28680 : CLRRKLEESSQQMQGQMWEQHAANLLGYDQLRQSPHQQQAPHHGGNGFFHPLDPTTEPTLQIGYTQEQINNACVAASFMPTWLP : 252
5B02G28610 : CLRRKLEESSQQMQGQMWEQHAANLLGYDQLRQSPHQQQATHHGGNGFFHPLDPTTEPTLQIGYTQEQINNACVAASFMPTWLP : 252
5D02G29450 : CLRRKLEESSQQMQGQMWEQHAANLLGYDQLRQSPHQQQAPHHGGNGFFHPLDPTTEPTLQIGYTQEQINNACVAASFMPTWLP : 252
```

Figure S5. Alignment of the three SEP3, OsMADS8/24-like homeolog proteins of *Triticum aestivum* (bread wheat).
